# Theoretical Analysis of Terahertz Frequency Multiplier Based on Semiconductor Superlattices

**DOI:** 10.3390/nano12071114

**Published:** 2022-03-28

**Authors:** Wei Feng, Shuting Wei, Yonghui Zheng, Chang Wang, Juncheng Cao

**Affiliations:** 1 School of Physics and Electronic Engineering, Jiangsu University, 301 Xuefu Road, Zhenjiang 212013, China; wfeng@ujs.edu.cn (W.F.); 2212026010@stmail.ujs.edu.cn (S.W.); 2 Center of Materials Science and Optoelectronics Engineering, University of Chinese Academy of Sciences, Beijing 100049, China; yhzheng@mail.sim.ac.cn; 3 Key Laboratory of Terahertz Solid-State Technology, Shanghai Institute of Microsystem and Information Technology, Chinese Academy of Sciences, 865 Changning Road, Shanghai 200050, China

**Keywords:** terahertz, multiplier, superlattice, magnetic field, balance equation approach

## Abstract

We propose a terahertz frequency multiplier based on high order harmonic generation in a GaAs-based miniband superlattice driven by an electric field. The performance of the frequency multiplier is analyzed using the balance equation approach, which incorporates momentum and energy relaxation processes at different lattice temperatures. It is found that the generated high-order harmonic power is sensitive to temperature changes. The peak power appears around resonance between driving terahertz frequency and intrinsic Bloch frequency. In the presence of the magnetic field, the peak power shifts towards a stronger static electric field region. The simulated results about the dependence of the second and third harmonic powers on a DC electric field are in qualitative consistence with the experiments. The proposed terahertz frequency multiplier based on semiconductor superlattice, being compact and efficient, is provided as a good candidate for terahertz wave generation.

## 1. Introduction

Semiconductor superlattice [1] is a periodical structure with layers of two or more semiconductor materials with similar lattice constants. During the past few decades, in semiconductor superlattice, many interesting phenomena have been observed, including DC current suppression with high frequency voltage bias [2,3], coherent Hall effect under the crossed electric and magnetic fields [4], periodic and chaotic electron transport under DC+AC electric field [5,6], etc. These phenomena are relevant to the nonlinearity of an electronic system and have provided abundant opportunities to develop compact sources and a spectroscopic scheme in the ‘terahertz gap’. The underlying mechanism of them is negative differential mobility (NDM), resulting from Bragg scattering of electrons at the boundary of the Brillouin zone.

Over the past twenty years, terahertz technology has attracted lots of interest due to its wide and important applications, such as nondestructive testing [7], imaging [8,9,10], high speed communication [11,12,13], and so on. Terahertz sources play a crucial role in these applications. The approaches for developing terahertz sources include optical terahertz generation, terahertz quantum cascade laser (THz QCL), and solid-state electronic devices [14]. Tunesi et al. [15] have reported that ultrafast metallization excited by a dual pump optical setup can generate a terahertz wave and, through this framework, a complex time-dependent photocarrier dynamics can be observed in black silicon. These newly developed optical terahertz generation schemes can create impact in complex nonlinear imaging and in the optical control of electron–electron interactions. THz QCL [16,17,18,19,20] operating at 2–5 THz has proved to be effective, compact, and coherent. However, the shortcoming is that THz QCL still needs to operate at liquid helium temperature to obtain excellent working performance. By comparison, the superlattice oscillator [21] can radiate microwaves at room temperature due to negative differential conductance (NDC) [22]. The radiation frequency can extend to the millimeter wave region by reducing the period of superlattice. In addition, within a certain range of the applied voltage, the current oscillations within the superlattice can be self-synchronized leading to a dramatic rise of the output power [23].

Recently, the superlattice harmonic multiplier has been proposed to generate millimeter and terahertz waves. Many theoretical [2,24] and experimental works [25,26,27,28] have been devoted to investigating the current–voltage (I–V) characteristics and the output power of the harmonic multiplier. Winnerl et al. [25] have reported frequency doubling and tripling of terahertz radiation in a voltage-biased GaAs/AlAs superlattice by taking into account elastic scattering and strong losses due to impedance mismatch among the mesa elements in their experiment. Apostolakis et al. [27] found that asymmetric current flow caused by static bias or interface roughness can generate even harmonics, and high order nonlinear output is not proportionate to the increasing input power. In this case, up to the 6th harmonic from the superlattice frequency multiplier can be realized at room temperature [28]. Moreover, strong terahertz gain in the superlattice oscillators can be achieved by avoiding space-charge instabilities if driving frequency reaches terahertz range [29,30].

Despite the rapid progress in fundamental sciences of superlattices and its application in terahertz sources, some key issues regarding the nature and performance of frequency multiplier based on miniband superlattice driven by electric and magnetic fields remain less understood. In this paper, we theoretically study the performance of the superlattice frequency multiplier by applying the balance equation approach. It is found that increasing lattice temperature can suppress the output power. The distribution of output power peak for frequency doubling and tripling is contributed from resonance between AC frequency and Bloch frequency. The paper is organized as follows: in Section 2, we give the semiclassical transport model describing electron dynamics in the superlattice frequency multiplier. The numerical results of high order harmonic coefficient and output power are presented in Section 3. The effects of temperature, external electric, and magnetic fields on the emission power of frequency multiplier are carefully analyzed. In Section 4, we summarize our work.

## 2. Balance Equation Approach for Electron Response of a Superlattice Frequency Multiplier

In this section, we consider the motion of electrons along the growth direction (*z*-axis) in the miniband of a GaAs-based superlattice. The superlattice is driven by an external terahertz radiation and a magnetic field *B*. The motion of electrons is confined in the lowest miniband and the inter-miniband tunneling effect is ignored. Within the tight-binding approximation, the electron energy dispersion of semiconductor superlattice can be written in the form

(1) $$\varepsilon \left(k\right)=\frac{\hbar^{2}\left(k_{x}^{2}+k_{y}^{2}\right)}{2m^{*}}+\frac{\Delta }{2}\left[1-\cos\left(k_{z}d\right)\right],$$

where *d* is the period of superlattice, $k=(k_{x},k_{y},k_{z})$ is the electron wave vector, $-\pi /d<k_{z}\leq \pi /d$ is the longitudinal (along the superlattice axis) components of wave vector, $m^{*}$ is the electron effective mass of GaAs, Δ is miniband width, and *ℏ* is the Plank constant.

Now, we apply an external electric field parallel to the growth direction of superlattice (*z* axis) and a magnetic field perpendicular to it, i.e., along the *x*-axis. The electrons confined in the miniband are accelerated by the applied fields and scattered by disorders and phonons, leading to an overall drift motion and heating of the electron system. The macroscopic average states of the superlattice can be described by the momentum and energy balance equations, in which the average drift velocity and average electron energy are both functions of the external fields. The balance equations for electron motions in the miniband are in the form [31,32,33,34]

(2) $$\frac{d\upsilon_{\mathrm{dr}}}{dt}=\frac{e\left[E\left(t\right)-B\upsilon_{y}\right]}{m_{z}^{*}\left(h_{ez}\right)}-\frac{\upsilon_{\mathrm{dr}}}{\tau_{\upsilon }},$$

(3) $$\frac{d\upsilon_{y}}{dt}=\frac{eB\upsilon_{\mathrm{dr}}}{m^{*}}-\frac{\upsilon_{y}}{\tau_{\upsilon }},$$

(4) $$\frac{dh_{e}}{dt}=eE\left(t\right)\upsilon_{\mathrm{dr}}-\frac{h_{e}-h_{e0}}{\tau_{\varepsilon }},$$

where $\upsilon_{y}$ and $\upsilon_{\mathrm{dr}}$ are electron average velocities along the *y*- and *z*-axis, respectively. $h_{e}=h_{ez}+\frac{1}{2}m^{*}\upsilon_{y}^{2}$ is electron average energy from semiclassical picture with $h_{ez}$ the average energy along the *z*-axis. *e* is the electron charge, $\tau_{\upsilon }$ is the momentum relaxation time, and $\tau_{\varepsilon }$ is the energy relaxation time. $m_{z}^{*}\left(h_{ez}\right)$ is the energy-dependent effective mass of electrons in the miniband and expressed as

(5) $$m_{z}^{*}\left(h_{ez}\right)=\frac{M_{0}}{1-2h_{ez}/\Delta },$$

with $M_{0}=2\hbar^{2}/\Delta d^{2}$ being the longitudinal effective mass at the bottom of the miniband.

Within the framework of the balance equation approach, the microscopic transport state of electron system is described by the center-of-mass momentum $p_{d}$ and the relative electron temperature $T_{e}$ . The ensemble-averaged quantities $\upsilon_{\mathrm{dr}}$ , $\upsilon_{y}$ , and $h_{ez}$ are functions of $p_{d}$ and $T_{e}$ . The average energy is written by $h_{ez}=\frac{\Delta }{2}\left[1-\alpha_{1}\left(T_{e}\right)\cos\left(p_{d}d/\hbar \right)\right]$ , where the expression of $\alpha_{1}\left(T_{e}\right)$ can be found in Ref. [34]. The thermal equilibrium energy is $h_{e0}=\frac{\Delta }{2}\left[1-\alpha_{1}\left(T\right)\right]$ with *T* the lattice temperature. In the Boltzmann’s distribution limit, we have $\alpha_{1}\left(T\right)=I_{1}\left(\Delta /2k_{B}T\right)/I_{0}\left(\Delta /2k_{B}T\right)$ with $k_{B}$ the Boltzmann’s constant, $I_{0}$ and $I_{1}$ the modified Bessel functions of the zeroth and first order, respectively. Thus, the thermal equilibrium energy $h_{e0}$ will be

(6) $$h_{e0}=\frac{\Delta }{2}\left[1-\frac{I_{1}\left(\Delta /2k_{B}T\right)}{I_{0}\left(\Delta /2k_{B}T\right)}\right].$$

The applied time-dependent electric field along the growth direction of the superlattice is $E\left(t\right)=E_{\mathrm{dc}}+E_{\mathrm{ac}}\cos\left(2\pi f_{\mathrm{ac}}t\right)$ , where $E_{\mathrm{dc}}$ is the DC electric field, $E_{\mathrm{ac}}$ and $f_{\mathrm{ac}}$ are the amplitude and frequency of an AC electric field, respectively. $f_{\mathrm{ac}}$ is in the region of terahertz frequency. By solving Equations (2)–(4), the drift velocity $\upsilon_{\mathrm{dr}}$ of electrons and average energy $h_{e}$ can be obtained, and we find that they get into steady time-periodic state after a short transient process. In steady state, the drift velocity $\upsilon_{\mathrm{dr}}$ and average energy $h_{e}$ are both periodic functions with $T_{\mathrm{ac}}=1/f_{\mathrm{ac}}$ . To analyse DC and harmonic characteristics of the superlattice, we make a Fourier transform of drift velocity $\upsilon_{\mathrm{dr}}$ , expressed by

(7) $$\upsilon_{\mathrm{dr}}\left(t\right)=\upsilon_{0}+\sum_{n=1}^{\infty }\left[\upsilon_{ns}\sin\left(n\omega t\right)+\upsilon_{nc}\cos\left(n\omega t\right)\right],$$

where $\upsilon_{0}$ is the DC component, n=1,2,3… is a positive integer, and $\upsilon_{ns}$ and $\upsilon_{nc}$ are the coefficients of sine component and cosine component for the *n*th harmonics, respectively. They are given by

(8) $$\upsilon_{0}=\frac{1}{T_{\mathrm{ac}}}\int_{0}^{T_{\mathrm{ac}}}\upsilon_{\mathrm{dr}}\left(t\right)dt,$$

(9) $$\upsilon_{ns}=\frac{2}{T_{\mathrm{ac}}}\int_{0}^{T_{\mathrm{ac}}}\upsilon_{\mathrm{dr}}\left(t\right)\sin\left(n\omega t\right)dt,$$

(10) $$\upsilon_{nc}=\frac{2}{T_{\mathrm{ac}}}\int_{0}^{T_{\mathrm{ac}}}\upsilon_{\mathrm{dr}}\left(t\right)\cos\left(n\omega t\right)dt.$$

The current density of the miniband superlattice under terahertz electric field can be written as $J\left(t\right)=eN\upsilon_{\mathrm{dr}}$ , with *N* being the electron concentration. It is proportional to the average velocity $\upsilon_{\mathrm{dr}}$ . Therefore, the current of high-order harmonics, defined as $j_{n}\left(t\right)=eN\left[\upsilon_{ns}\sin\left(n\omega t\right)+\upsilon_{nc}\cos\left(n\omega t\right)\right]$ , indicates that the miniband superlattice can operate as a high frequency multiplier.

## 3. Performance Analysis of the Superlattice Terahertz Frequency Multiplier

In this section, with the above balance equation approach, we study the effect of electromagnetic field on the performance of the superlattice frequency multiplier. Here, a GaAs-based n-type superlattice with period d=4.8nm , miniband width ▵=70meV , and the density of carriers $N=1.07\times 10^{18}\;{\mathrm{cm}}^{-3}$ is considered. We calculate the output power of superlattice at lattice temperature 4.2K , 77K , and 300K , respectively. In the calculations, the momentum relaxation time $\tau_{\upsilon }$ and energy relaxation time $\tau_{\varepsilon }$ are not constant. They are functions of average electron energy $h_{ez}$ [34].

**Figure 1:**
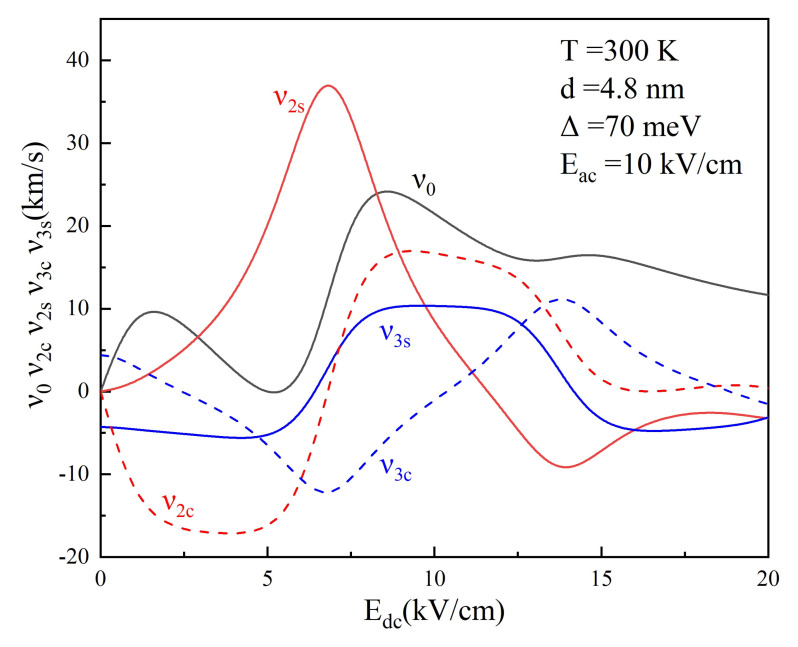
DC component $\upsilon_{0}$ and harmonic coefficients $\upsilon_{2s},\upsilon_{2c},\upsilon_{3s}$ , and $\upsilon_{3c}$ of drifty velocity $\upsilon_{\mathrm{dr}}$ are plotted as a function of $E_{\mathrm{dc}}$ in GaAs-based superlattice driven by $E_{\mathrm{ac}}=10\;\mathrm{kV}/\mathrm{cm}$ at T=300K . Other superlattice parameters are d=4.8nm and Δ=70meV .

First, we investigate the high order harmonic generation and emitted harmonic power of semiconductor superlattice under the terahertz electric field and in the absence of magnetic field *B*. Figure 1 shows the DC component $\upsilon_{0}$ , the second and third harmonic coefficients of sine component and cosine component $\upsilon_{2s}$ , and $\upsilon_{2c}$ , $\upsilon_{3s}$ , $\upsilon_{3c}$ of drift velocity in the superlattice. The lattice temperature is T=300K , and the frequency of the applied electric field is 0.8THz . From the figure, we can find that second harmonic coefficients $\upsilon_{2s}$ , $\upsilon_{2c}$ vanish with DC bias $E_{\mathrm{dc}}=0\;\mathrm{kV}/\mathrm{cm}$ . In other words, the superlattice frequency multiplier can only achieve odd harmonic generation with DC bias $E_{\mathrm{dc}}=0\;\mathrm{kV}/\mathrm{cm}$ . Although the fluctuation of second harmonic coefficients is much stronger compared to the third harmonic with $E_{\mathrm{dc}}$ increasing, the oscillation amplitudes of the second and third harmonics are relatively large. This implies that semiconductor superlattice can be used as a terahertz frequency multiplier with considerable power emission.

By Electromagnetic Theory, the power of the emitted *n*th harmonic $P_{n}\left(\omega \right)$ is defined as [27]

(11) $$P_{n}\left(\omega \right)=\frac{A\mu_{0}cL^{2}}{8n_{r}}I_{n}\left(\omega \right),$$

where A=100 μ m ${}^{2}$ is the mesa area of a superlattice, and $\mu_{0}$ and *c* are the permeability and the speed of light, respectively. *L* is effective path length through the superlattice, and $n_{r}=\sqrt{13}$ is the refractive index of GaAs. $I_{n}\left(\omega \right)$ is the root-mean-square value of the *n*th component of the Fourier expansion of the induced current density written as

(12) $$I_{n}\left(\omega \right)={\langle j_{n}\left(t\right)\cos\left(n\omega t\right)\rangle }_{t}^{2}+{\langle j_{n}\left(t\right)\sin\left(n\omega t\right)\rangle }_{t}^{2},$$

where ${\langle \ldots \rangle }_{t}$ is performed as an integration over the period $T_{\mathrm{ac}}$ .

**Figure 2:**
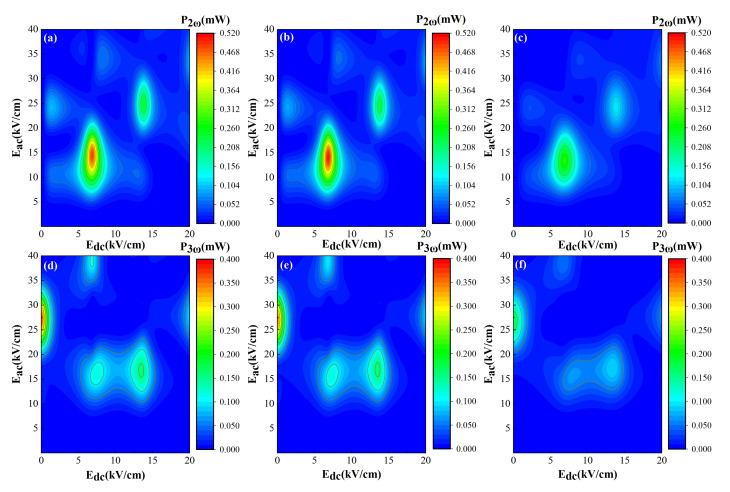
The calculated second harmonic output powers (**a**–**c**) and third harmonic ones (**d**–**f**) as functions of $E_{\mathrm{dc}}$ and $E_{\mathrm{ac}}$ . The harmonic power is generated by a GaAs-based superlattice with d=4.8nm driven by an AC electric field with frequency $f_{\mathrm{ac}}=0.8\;\mathrm{THz}$ . The lattice temperatures are as follows: (**a**,**d**) 4.2K , (**b**,**e**) 77K , and (**c**,**f**) 300K .

Now, we consider the emitted power of the second and third harmonics at different lattice temperatures T=4.2K , 77K , and 300K for the same driving frequency $f_{\mathrm{ac}}=0.8\;\mathrm{THz}$ . Figure 2a–c display the second harmonic power $P_{2}\left(\omega \right)$ generated by the superlattice frequency multiplier as functions of $E_{\mathrm{dc}}$ and $E_{\mathrm{ac}}$ . Figure 2d–f reveals the third harmonic power $P_{3}\left(\omega \right)$ as functions of $E_{\mathrm{dc}}$ and $E_{\mathrm{ac}}$ . The lattice temperatures are as follows: Figure 2a,d 4.2K , Figure 2b,e 77K , and Figure 2c,f 300K . The red and orange areas indicate higher output power, while the blue zone represents a lower one. From Figure 2a–c, we can see that, at T=4.2K , 77K , and 300K , the power maxima of the second harmonic are 0.485mW , 0.556mW , and 0.275mW , respectively. With lattice temperature increasing to room temperature T=300K , the maximum power of the second harmonic is reduced, while the zone of output power is only slightly expanded. This tendency results from the decrease of electron drift velocity with the increase of the temperature. It is interesting that these maxima all appear at the same $E_{\mathrm{dc}}=6.86\;\mathrm{kV}/\mathrm{cm}$ and $E_{\mathrm{ac}}=14.48\;\mathrm{kV}/\mathrm{cm}$ regardless of the temperature. With the Bloch frequency $f_{B}=eE_{\mathrm{dc}}d/\left(2\pi \hbar \right)$ , we further find that these peaks locate at exactly where $f_{B}=f_{\mathrm{ac}}$ and $f_{B}=2f_{\mathrm{ac}}$ . The maximum power originates from resonance between driving terahertz AC frequency and intrinsic Bloch frequency. Figure 2d–f shows emitted power $P_{3}\left(\omega \right)$ of the third harmonic at 4.2K , 77K , and 300K , respectively. The corresponding maximum values of third harmonic power are 0.363mW , 0.344mW , and 0.169mW . Similar to the second harmonic, the maximum power decreases as the temperature increases. However, different from the second harmonic, in the power diagram of $P_{3}\left(\omega \right)$ , locations of the main peaks are at $E_{\mathrm{dc}}=0$ and $E_{\mathrm{ac}}=27.24\;\mathrm{kV}/\mathrm{cm}$ . The appearance at $E_{\mathrm{dc}}=0$ indicates that odd harmonic coefficients play a dominant role in the frequency tripler in the absence of a static electric field. Furthermore, by comparison of Figure 2a and Figure 2b, Figure 2b and Figure 2e, or Figure 2c and Figure 2f, we can conclude that the higher the harmonic order is, the weaker the output power exhibits. Thus, it is reasonable that we will not take the higher order harmonic radiation with n>3 into consideration in our study.

**Figure 3:**
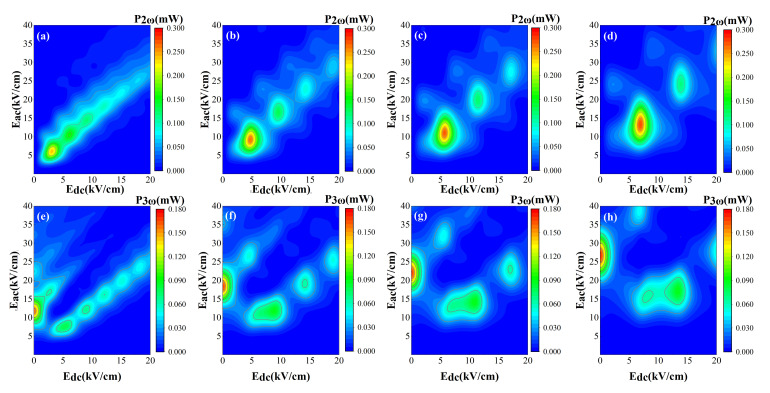
The second and third harmonic output powers as functions of $E_{\mathrm{dc}}$ and $E_{\mathrm{ac}}$ . The period of superlattice is d=4.8nm and temperature is T=300K . The AC electric field frequencies $f_{\mathrm{ac}}$ presented in figure are defined as follows: (**a**,**e**) 0.35THz , (**b**,**f**) 0.55THz , (**c**,**g**) 0.66THz , and (**d**,**h**) 0.8THz .

Since working at room temperature is an important feature of terahertz devices, we now focus on the harmonic generation of semiconductor superlattice at T=300K . Further insight on how driving frequency can affect the location of harmonic peak power is given by the color contour plot in Figure 3. Here, the driving frequencies are set as $f_{\mathrm{ac}}=0.35\;\mathrm{THz}$ , 0.55THz , 0.66THz , and 0.8THz . Figure 3 shows that, by increasing the parameter $f_{\mathrm{ac}}$ , the emitted maximum power of $P_{2}\left(\omega \right)$ and $P_{3}\left(\omega \right)$ increase slowly. The power distribution of $P_{2}\left(\omega \right)$ is shown in Figure 3a–d. The maximum of emitted power also occurs at the resonance between AC field $f_{\mathrm{ac}}$ and Bloch frequency $f_{B}$ . For example, Figure 3a displays that peaks of the second harmonic power appear at $E_{\mathrm{dc}}≃3.01\;\mathrm{kV}/\mathrm{cm}$ , 6.03kV/cm , 9.04kV/cm , and 12.05kV/cm , etc. With the increase of DC bias $E_{\mathrm{dc}}$ or AC field $E_{\mathrm{ac}}$ , the magnitude of the peaks drops progressively. In particular, the distribution of the peaks shows a linear function dependence on $E_{\mathrm{dc}}$ and $E_{\mathrm{ac}}$ . Figure 3e–h depict the power distribution of $P_{3}\left(\omega \right)$ , whose output power peak distribution is more complex and irregular than that of $P_{2}\left(\omega \right)$ . The maximum power peak of $P_{3}\left(\omega \right)$ appears at $E_{\mathrm{dc}}=0$ . As driving frequency increases, we observe that the power maximum of $P_{3}\left(\omega \right)$ appears at a stronger AC electric field, ranging from $E_{\mathrm{ac}}=11.82\;\mathrm{kV}/\mathrm{cm}$ to $E_{\mathrm{ac}}=26.70\;\mathrm{kV}/\mathrm{cm}$ in Figure 3e–h. The other peak distributions of $P_{3}\left(\omega \right)$ are not directly related to $f_{B}=if_{\mathrm{ac}}\left(i=1,2,3\ldots \right)$ , which is different from that of the second harmonic. Figure 3e,f manifest that the magnitude of the secondary peak appearing at a small value of ( $E_{\mathrm{dc}}$ , $E_{\mathrm{ac}}$ ) is half of the maximum peak. When driving frequency becomes large, as shown in Figure 3h for $f_{\mathrm{ac}}=0.8\;\mathrm{THz}$ , the secondary peak splits into two with peak powers appearing at $E_{\mathrm{ac}}=14.28\;\mathrm{kV}/\mathrm{cm}$ and $E_{\mathrm{ac}}=17.25\;\mathrm{kV}/\mathrm{cm}$ , respectively.

**Figure 4:**
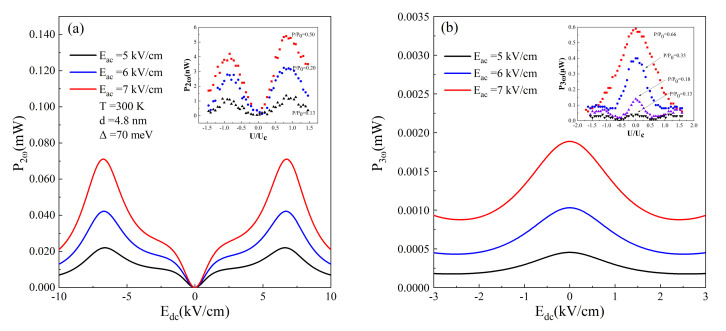
(**a**) Second and (**b**) third harmonic output powers are plotted as functions of the DC electric field $E_{\mathrm{dc}}$ . The applied AC electric field is $E_{\mathrm{ac}}=5\;\mathrm{kV}/\mathrm{cm}$ (black line), 6kV/cm (red line), and 7kV/cm (blue line), respectively. The lattice temperature is T=300K and the AC frequency is $f_{\mathrm{ac}}=0.8\;\mathrm{THz}$ . The insets of (**a**,**b**) describe the experimental results of the second and third harmonic power, respectively, versus normalized voltage for different excitation power that is proportional to $E_{\mathrm{ac}}^{2}$ . Reprinted with permission from Ref. [25]. Copyright 2000 American Institute of Physics.

To focus on the characteristic of $P_{n}\left(\omega \right)$ versus static electric field, we show the emitted power profile of high order harmonic in Figure 4. Three different AC electric fields $E_{\mathrm{ac}}=5\;\mathrm{kV}/\mathrm{cm}$ , 6kV/cm , and 7kV/cm are applied at room temperature with the same frequency $f_{\mathrm{ac}}=0.8\;\mathrm{THz}$ . Despite the AC electric fields, the peak power appears at the same value of $E_{\mathrm{dc}}$ , where driving AC frequency is in resonance with Bloch frequency. The experimental demonstration of frequency doubling and tripling with semiconductor superlattice frequency multiplier has been reported in Ref. [25]. The superlattice consisting of 100 periods is grown by molecular beam epitaxy on a GaAs substrate. A corner cube antenna system can be fabricated to guide the terahertz radiation from a free-electron laser into the mesa element of frequency multiplier. The generated second and third harmonics are coupled out of the mesa by the corner cube antenna. The doping density of superlattice layers is $N=5\times 10^{17}\;{\mathrm{cm}}^{-3}$ . The experimental results of the second and third harmonic powers are reproduced in the insets of Figure 4. Our calculations of $P_{2}\left(\omega \right)$ and $P_{3}\left(\omega \right)$ are in qualitative consistence with the experiments [25]. It is noted that the experimental power is smaller by several orders and this may be attributed to light doping density and device loss in measurement of the experiment.

**Figure 5:**
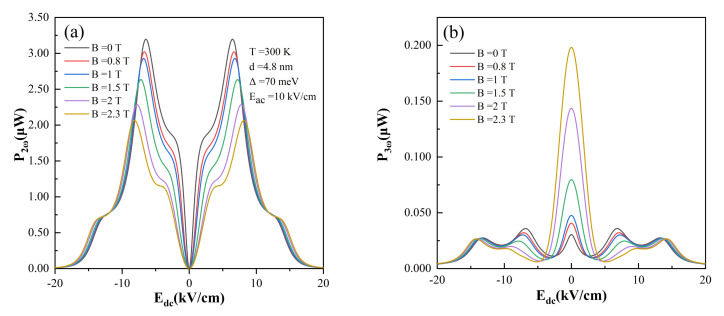
(**a**) Second and (**b**) third harmonic output powers are plotted as functions of the DC electric field $E_{\mathrm{dc}}$ . The applied perpendicular magnetic fields are B=0T (black line), 0.8T (red line), 1T (blue line), 1.5T (green line), 2T (purple line), and 2.3T (yellow line), respectively. The lattice temperature is T=300K and the AC frequency is $f_{\mathrm{ac}}=0.8\;\mathrm{THz}$ .

Finally, we demonstrate that the high order harmonic output power can be tuned by introducing a magnetic field perpendicular to the growth axis. Figure 5a,b exhibit $P_{2}\left(\omega \right)$ and $P_{3}\left(\omega \right)$ , respectively, as functions of $E_{\mathrm{dc}}$ . The applied AC electric field is set to be constant as $E_{\mathrm{ac}}=3\;\mathrm{kV}/\mathrm{cm}$ while the magnetic fields are different as B=0 , 0.8T , 1T , 1.5T , 2T , and 2.3T . The superlattice miniband width is Δ=70meV and lattice temperature is still T=300K . The AC frequency is $f_{\mathrm{ac}}=0.8\;\mathrm{THz}$ . Illustrated from Figure 5, the harmonic output power peak shifts to a stronger DC electric field with the magnetic field increasing. It can be understood by the fact that electrons perform cyclotron oscillation with frequency $f_{c}=\frac{eB}{m^{*}}$ , which increases with magnetic field and corresponds to a stronger DC electric field. Interestingly, the harmonic power profile reveals that the magnetic field has no effect on the lineshape distribution, while magnitude of harmonic power is sensitive to the magnetic field. Thus, two-dimensional images rather than contour plots are used here. It is worth noting that, as shown in Figure 5b, output power enhances along with the increase of the magnetic field at $E_{\mathrm{dc}}=0\;\mathrm{kV}/\mathrm{cm}$ not being universal, and this rule is broken at other $E_{\mathrm{ac}}$ values. On the whole, the magnetic field has an inhibitory effect on emitted harmonic power.

## 4. Conclusions

In summary, using the balance equation approach with relaxation time depending on lattice temperature and miniband width, we theoretically analyze harmonic coefficients of drift velocity and emitted harmonic power of a semiconductor superlattice frequency multiplier in terahertz frequency range. It is found that high lattice temperature can suppress the peak of $P_{n}\left(\omega \right)$ and result in enhancement of the peak width. Meanwhile, the peak of the second harmonic output power $P_{2}\left(\omega \right)$ appears at the resonance between driving frequency and Bloch frequency. The peak distribution of $P_{3}\left(\omega \right)$ is much more complicated except that the emission power maximum is dominant at $E_{\mathrm{dc}}=0\;\mathrm{kV}/\mathrm{cm}$ . With the presence of the magnetic field, the peak position of emitted power can be tuned due to cyclotron oscillation. Our simulations are in good agreement with the experiments, which verifies the validity of the balance equation approach. The compact and efficient terahertz frequency multiplier based on a semiconductor superlattice is proposed to be a good candidate for terahertz wave generation.

## Data Availability

The data that support the findings of this study are available from the corresponding author upon reasonable request.
